# Prognostic Value of Rab27B Nuclear Expression in Gastrointestinal Stromal Tumors

**DOI:** 10.1155/2014/942181

**Published:** 2014-10-14

**Authors:** Wei Wang, Qichao Ni, Hua Wang, Shu Zhang, Huijun Zhu

**Affiliations:** ^1^Department of Pathology, Nantong University Affiliated Hospital, Nantong, Jiangsu 226001, China; ^2^Department of General Surgery, Nantong University Affiliated Hospital, Nantong, Jiangsu 226001, China

## Abstract

Rab proteins of the endocytosis and exocytosis pathways both play critical roles in cancer progression, and Rab27B has a significant relationship with several types of human cancer. However, the association between Rab27B expression and clinical features to determine its clinicopathological significance in gastrointestinal tumor (GIST) has not been investigated. To examine the expression of Rab27B in GIST and investigate the association between its expression and patient prognosis, immunohistochemistry analysis with tissue microarray was used to evaluate expression of Rab27B in 162 patients with GIST. The relationship between Rab27B expression and patient prognosis was analyzed. High nuclear staining of Rab27B was detected in 88 of 162 (54.32%) GIST tissues. Positive staining of Rab27B was significantly associated with tumor size (*P* = 0.006), mitotic index (*P* = 0.013), Armed Forces Institute of Pathology Miettinen risk classification (*P* = 0.002), and tumor grade (*P* = 0.021). Kaplan-Meier survival curves showed that GIST patients with low Rab27B nuclear expression (*P* = 0.038) and mitotic index <5 per 50 high-power fields (*P* = 0.029) had a more favorable prognosis. These findings indicate that Rab27B nuclear expression is correlated with several clinicopathological features of GIST patients, and it may serve as an unfavorable prognostic marker.

## 1. Introduction

Gastrointestinal stromal tumor (GIST) is the most common primary mesenchymal tumor that arises from the gastrointestinal tract, with an annual incidence of 10–15 cases per million [1, 2]. The majority of GISTs develop in the stomach (60%), small bowel (30%), esophagus, and rectum (10%) [3]. GIST is a malign disease that ranges from a curable disorder to a highly malignant disease. GISTs are recognized immunohistochemically by CD117, the 145 kDa transmembrane glycoprotein KIT and a receptor for stem cell factor [4]. GISTs are a large set of tumors with a different spectrum of clinical characteristics and variable malignant potential, making it unique to distinguish between benign and malignant lesions. Mitotic index, tumor size, and tumor location, which are well-known prognostic predictors for GIST patients, form the basis of risk-stratification schemes that have been developed for operable GIST, including the National Institutes of Health Fletcher criteria and the Armed Forces Institute of Pathology (AFIP) Miettinen criteria [5–7].

For now, surgery is the gold standard for the treatment of localized primary GIST without metastasis [8], and imatinib mesylate (IM), which is a receptor tyrosine kinase inhibitor, is reportedly effective in patients with metastatic GIST, and adjuvant imatinib treatment improves GIST prognosis dramatically [9, 10]. However, the potential toxic effects and financial cost of this treatment, especially in poor areas in China, are substantial. Therefore, predicting the recurrence risk and malignancy potential of GISTs is of great importance and worth further exploration.

Recently, a large body of evidence has shown that vesicle trafficking and exocytosis are essential in tumorigenesis, which implicates the Rab family proteins [11–13]. Rab family proteins, which comprise >60 mammalian members and are thought to localize to a distinct subcellular organelle, are a ubiquitously expressed family of small (20–29 kDa) monomeric Ras-like GTPases [14]. The secretory Rab GTPases, involving Rab26, Rab37, Rab3A/B/C/D, and Rab27A/B, are reported to be fundamental for regulated vesicle exocytosis. Among these various GTPases, the homologs Rab27A and Rab27B constitute the Rab27 subfamily and are 71% identical at the amino acid level [15]. Rab27A causes the human hereditary disease, type 2 Griscelli syndrome, which is characterized by silvery hair and immunodeficiency (defects in granule exocytosis by cytotoxic T lymphocytes) [16]. However, presumably because of the compensatory effect of Rab27B, it has been reported that there is no abnormality in secretory cells other than the cytotoxic T lymphocytes in type 2 Griscelli syndrome patients. Actually, Rab27B is often expressed in various secretory cells along with Rab27A [17] and has been estimated to have the ability to bind all of the Rab27A effectors [18]. Recently, it has been indicated that a Rab27-effector system is involved in primary types of constitutive secretion by nonsecretory cells [19], demonstrating that Rab27B is a more general regulator in secretory pathways.

Rab proteins of the endocytosis and exocytosis pathways both play critical roles in cancer progression, and a few studies have investigated the role of Rab27B in several types of human cancer [14, 15, 20–23]. However, the association between Rab27B expression and clinical features, to determine its clinicopathological significance in GIST, has not been investigated. The potential of Rab27B as a candidate for molecular-targeted therapy of GIST requires further exploration.

In this study, we investigated expression of Rab27B in a selected group of GIST tissue samples. We analyzed the association between Rab27B expression and clinicopathological attributes in GIST patients. Finally, we explored the prognostic characteristics of Rab27B protein expression in GIST.

## 2. Materials and Methods

### 2.1. Patient Samples

We enrolled 162 GIST patients from the Department of Pathology, Nanjing First Hospital Affiliated to Nanjing Medical University and the Affiliated Hospital of Nantong University, between 2003 and 2010. Diagnosis of GIST was confirmed by positive immunohistochemical staining for c-KIT and was in accord with histopathological characteristics of GIST. Other clinical data included patient age, tumor size, mitotic index, gross classification, tumor location, tumor risk classification, and tumor grade. The potential risk classification for malignancy was evaluated using AFIP Miettinen risk classification criteria [6, 24]. None of the patients received preoperative radiotherapy or chemotherapy. Written informed consent was acquired from each patient for publication of this study, and the research protocol was approved by the human research ethics committee of each hospital.

### 2.2. Tissue Microarray (TMA) Construction and Immunohistochemical (IHC) Analysis

A total of 162 formalin-fixed, paraffin-embedded GIST tissues, collected between 2003 and 2010, were obtained from the Nanjing First Hospital Affiliated to Nanjing Medical University and Affiliated Hospital of Nantong University. We used the Tissue Microarray System (Quick-Ray, UT06; UNITMA, Korea) in the Department of Clinical Pathology, Nantong University Hospital, Jiangsu, China. Core tissue biopsies (2 mm in diameter) were taken from individual paraffin-embedded sections and arranged in recipient paraffin blocks as described previously [25].

IHC analysis was executed to detect the protein expression of Rab27B in GIST. Paraffin tissue sections (4 *μ*m) were deparaffinized in 100% xylene and rehydrated in a descending ethanol series according to standard protocols. The TMAs were incubated for 1 h with primary anti-Rab27B antibody (1 : 100 dilution, ab104083; Abcam, Cambridge, MA, USA), washed, and incubated with an anti-rabbit horseradish-peroxidase-conjugated antibody. As a negative control, phosphate-buffered saline was used instead of the primary antibody. Rab27B immunostaining was scored by two pathologists according to the intensity and density of Rab27B-positive cells. Staining intensity was scored according to four grades: 0, 1, 2, or 3, ranging from negative and weak to strong intensity. The density of Rab27B-positive cells was also scored at four levels: 0 for 0–29%, 1 for 30–59%, 2 for 60–79%, and 3 for 80–100%. The product of the intensity and density scores was used as the final Rab27B staining score. The cutoff point for the Rab27B expression score that was statistically significant in terms of overall survival (OS) was set using the X-tile software program (Rimm Laboratory at Yale University; http://www.tissuearray.org/rimmlab/) as described previously [26]. The degree of Rab27B staining was quantified using a two-level grading system, and staining scores were defined as follows: 0 or 1: low expression and 2–9: high expression.

### 2.3. Statistical Analysis

The association between protein expression of Rab27B and clinicopathological attributes was analyzed by *χ* ^2^ tests. Survival rate was evaluated using the Kaplan-Meier method and compared using the log rank test. Univariate and multivariate analyses were performed using Cox proportional hazards regression models. All statistical analyses were conducted by SPSS version 18.0 (SPSS, Chicago, IL, USA) and STATA version 12.0 (StataCorp, College Station, TX, USA) statistical software.

## 3. Results

### 3.1. Clinical Attributes of GIST Patients

73 men and 89 women were enrolled in this study. 98 patients were aged <60 years and 62 patients were aged >60 years. 40 patients had tumors <5 cm in diameter, 78 had tumors 5–10 cm in diameter, and 36 had tumors >10 cm in diameter. As for mitotic index, 67 patients had 0–5, 54 had 5–10, and 26 had >10. 11 patients had single nodules, while 18 had multiple nodules. Tumors in 81 patients were in the stomach, 56 in the intestines, and 23 in other organs. 34 patients were in the very low to low risk group and 41 in the moderate to high risk group, as evaluated by AFIP Miettinen risk classification. 48 patients were grade 1, 60 were grade 2, 28 were grade 3, and 10 were grade 4.

### 3.2. Expression and Location of Rab27B in GISTs by IHC Analysis

**Figure 1:**
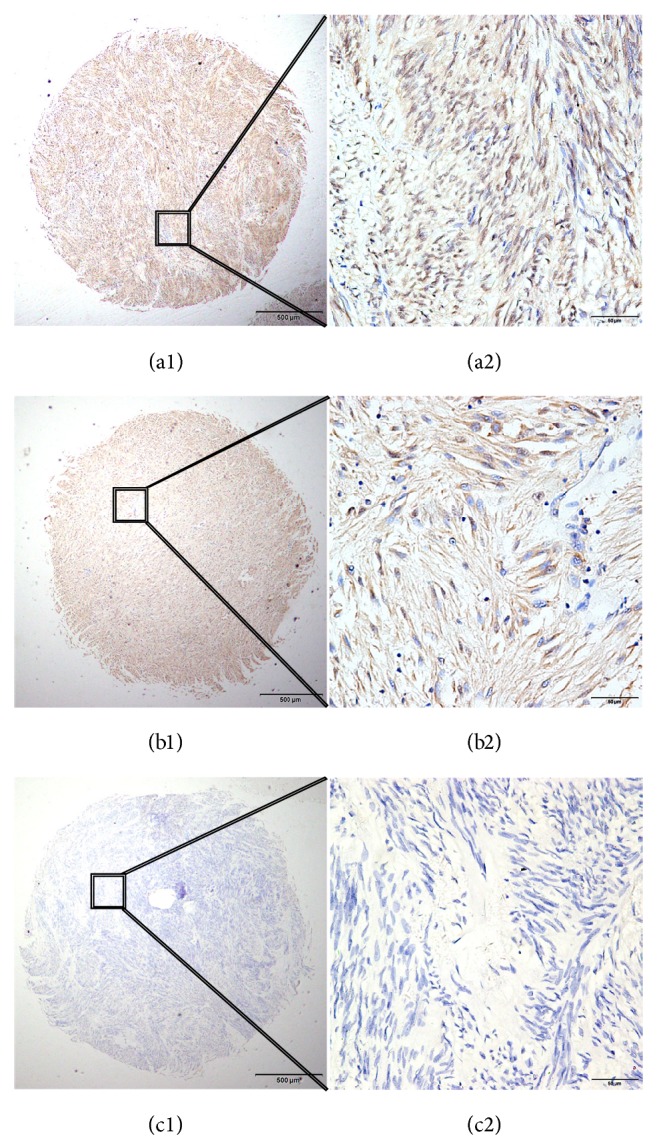
IHC staining of Rab27B in clinical tissue samples of GIST. ((a1), (a2)) High nuclear staining of Rab27B in GIST TMA samples. ((b1), (b2)) High cytoplasmic staining of Rab27B in GIST TMA samples. ((c1), (c2)) Negative staining for Rab27B in GIST TMA samples. Original magnification: ((a1), (b1), (c1)) ×40, ((a2), (b2), (c2)) ×400.

Expression of Rab27B in GIST TMAs was evaluated by IHC analysis. Representative Rab27B staining patterns are presented in Figure 1. Rab27B-positive staining was mainly localized to the nucleus of the tumor cells. All tissue samples were scored and categorized according to the cutoff point for Rab27B expression determined using the X-tile software program. In tumor cells, high Rab27B protein expression with nuclear staining was detected in 88 of 162 (54.32%) GIST tissues and the remaining 74 showed low or no Rab27B protein expression with nuclear staining. For 14.8% of the cases, positive staining of Rab27B was also detected in the cytoplasm of GIST cells (24 of 162).

### 3.3. Association between Rab27B Protein Expression and Clinicopathological Attributes

The association between Rab27B protein expression and the clinicopathological attributes of the 162 GIST patients is shown in Table 1. High nuclear staining of Rab27B was significantly associated with tumor size (*P* = 0.006), mitotic index (*P* = 0.013), AFIP Miettinen risk classification (*P* = 0.002), and tumor grade (*P* = 0.021). In contrast, Rab27B protein expression was not associated with other clinical parameters, including sex, age, gross classification, and tumor location (Table 1).

### 3.4. Survival Analysis

**Figure 2:**
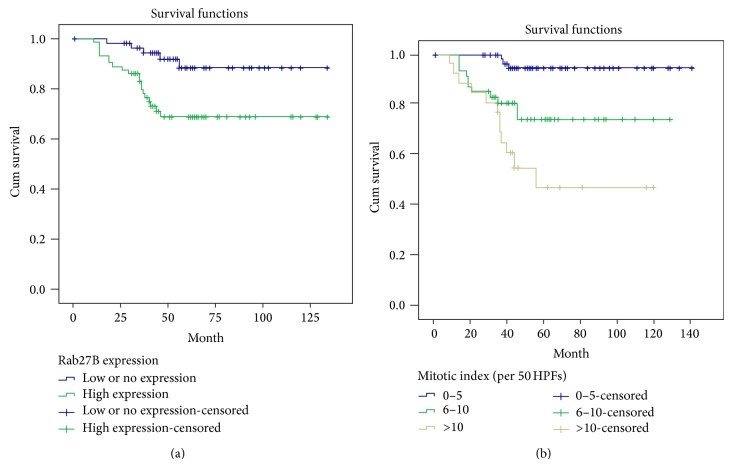
Kaplan-Meier analysis of the relationship between clinicopathological factors and OS of GIST patients. OS was significantly longer in patients with (a) low (blue line) or no versus high Rab27B expression (green line) and (b) low (blue line) versus large mitotic index (yellow and green line).

Univariate analyses showed that increased nuclear expression of Rab27B (*P* = 0.009), tumor diameter (*P* = 0.010), mitotic index (*P* < 0.001), AFIP Miettinen risk classification (*P* = 0.049), and tumor grade (*P* < 0.001) were associated with prognosis of GIST patients for 5-year OS rates (Table 2). Multivariate analyses further indicated that high nuclear expression of Rab27B (*P* = 0.038) and mitotic index (*P* = 0.029) were significantly correlated with 5-year OS rates (Table 2). Kaplan-Meier survival curves showed that GIST patients with low or no Rab27B nuclear expression and mitotic index <5 per 50 high-power fields had a more favorable prognosis (Figures 2(a) and 2(b)).

## 4. Discussion

It has been widely reported that Rab proteins of the endocytic pathway (e.g., Rab25, Rab13, Rab23, and Rab5) and the constitutive secretory pathway (e.g., Rab8) play significant roles in malignancy [20, 27–30]. Rab GTPases that regulate exocytosis (e.g., Rab27A and Rab37) could also be crucial for cancer progression [13, 31]. It has been reported that increased Rab27B expression correlates with the degree of cancer progression and might facilitate the invasive phenotypes. Rab27B has been suggested to function as an oncogene in breast cancer [32] because it is responsible for regulating many secretory mechanisms [15]. Furthermore, Rab27B has also been identified as a predictor of prognosis in certain human cancers, and high expression leads to unfavorable survival [14, 15]. All the above indicate the oncogenic characteristics of Rab27B; hence, we attempted to verify the relationship between Rab27B expression and various clinicopathological parameters of GIST.

By IHC analysis, we detected positive nuclear staining of Rab27B overexpression and 54.32% of GIST patients showed high nuclear staining for Rab27B. High nuclear staining for Rab27B was significantly related to tumor size, mitotic index, AFIP Miettinen risk classification, and tumor grade. There is one finding worth mentioning. For 14.8% cases, positive staining of Rab27B was also detected in the cytoplasm of GIST cells (24 of 162). However, there was no significant relationship between cytoplasmic expression of Rab27B and clinical parameters of GIST (data not shown). Although some studies have reported that positive staining of Rab27B is mainly localized in the cytoplasm of cancer cells [14, 15], we observed dominant nuclear staining of Rab27B in the present study. The inconsistencies may be due to the variance of tumor types and antibodies used. In addition, one deficiency of our IHC analysis was the absence of control groups, such as adjacent noncancerous tissue. In our previous research of cancer biomarkers, we usually used cancerous tissues for an experimental group and matched noncancerous tissues for a control group [25]. However, because of the specific nature of GIST, which originates from interstitial cells of Cajal or Cajal-like precursor cells, it is difficult to collect control group tissues from human samples because we cannot identify noncancerous tissues from tumor tissue samples. Hence, in our present IHC analysis, negative expression of Rab27B in whole TMA sections was set as an internal control, which was subsequently used for further comparison and analysis with other high and low Rab27B expression samples.

Univariate analysis indicated that low Rab27B expression was correlated with favorable prognosis of GIST patients in 5-year OS rates. Subsequent multivariate analysis revealed that high nuclear staining for Rab27B and high mitotic count were independent predictors of poor prognosis in GIST patients. These results are consistent with findings showing that upregulated Rab27B expression was detected and recognized as a poor prognostic factor [14, 15].

In conclusion, this study is believed to be the first to analyze Rab27B expression in GIST, and high Rab27B expression was correlated with several clinicopathological features of GIST. Rab27B may be a novel prognostic marker of GIST and Rab27B-based molecular-targeted therapy may provide a promising strategy for GIST treatment.

## Figures and Tables

**Table 1:** Association of Rab27B expression with clinical parameters of GIST.

Groups	Number	Rab27B			
		Low or no expression (%)	High expression (%)	Pearson *χ* ^2^	*P* value
Total	**162**	**74 (45.68)**	**88 (54.32)**		
Gender				0.012	0.913
Male	73	33 (45.21)	40 (54.79)		
Female	89	41 (46.07)	48 (53.93)		
Age				0.054	0.816
≤60 years	98	44 (44.90)	54 (55.10)		
>60 years	62	29 (46.77)	33 (53.23)		
Unknown	2	1	1		
Tumor size				10.228	0.006∗
<5 cm	40	21 (52.50	19 (47.50)		
5–10 cm	78	41 (52.56)	37 (47.44)		
≥10 cm	36	8 (22.22)	28 (77.78)		
Unknown	8	4	4		
Mitotic index (per 50 HPFs)				8.639	0.013∗
0–5	67	38 (56.72)	29 (43.28)		
6–10	54	24 (44.44)	30 (55.56)		
>10	26	6 (23.08)	20 (76.92)		
Unknown	15	6	9		
Gross classification				0.184	0.668
Single nodule	11	4 (36.36)	7 (63.64)		
Multiple nodules	18	8 (44.44)	10 (55.56)		
Unknown	133	62	71		
Tumor location				0.571	0.752
Stomach	81	37 (45.68)	44 (54.32)		
Intestine	56	24 (42.86)	32 (57.14)		
Others	23	12 (52.17)	11 (47.83)		
Unknown	2	1	1		
AFIP Miettinen risk classification				9.182	0.002∗
Very low-low risk	34	20 (58.82)	14 (41.18)		
Moderate-high risk	41	10 (24.39)	31 (75.61)		
Unknown	87	44	43		
Grade				9.703	0.021∗
1	48	30 (62.50)	18 (37.50)		
2	60	27 (45.00)	33 (55.00)		
3	28	8 (28.57)	20 (71.43)		
4	10	3 (30.00)	7 (70.00)		
Unknown	16	6	10		

^*^ *P* < 0.05; HPFs: high-power fields.

**Table 2:** Univariate and multivariate analysis of prognostic factors in GIST for 5-year survival.

	Univariate analysis				Multivariate analysis			
	HR	*P* > |*z*|	95% CI		HR	*P* > |*z*|	95% CI	
Rab27B expression								
High versus low	2.61	0.009∗	1.384	9.843	2.07	0.038∗	1.070	10.925
Gender								
Male versus female	−1.52	0.128	.250	1.191				
Age (years)								
≤60 versus >60	0.32	0.746	.531	2.423				
Tumor diameter (cm)								
<5 versus 5–10 versus ≥10	2.57	0.010∗	1.202	3.894				
Mitotic index (per 50 HPFs)								
0–5 versus 6–10 versus >10	4.37	<0.001∗	1.885	5.287	2.18	0.029∗	1.236	50.063
Gross classification								
Single versus multiple	0.47	0.639	.337	5.904				
Tumor position								
Stomach versus intestine versus others	0.28	0.782	.629	1.850				
AFIP Miettinen risk classification								
Very low-low risk versus moderate-high risk	1.97	0.049∗	1.003	8.178				
Tumor grade								
Stages I-II versus Stages III-IV	4.18	<0.001∗	1.560	3.415				

^*^ *P* < 0.05.
